# A Cross-Sectional Study for Prevalence and Association of Risk Factors of Chronic Kidney Disease Among People With Type 2 Diabetes in the Indian Setting

**DOI:** 10.7759/cureus.18371

**Published:** 2021-09-29

**Authors:** Ajoy Tewari, Vineeta Tewari, Jay Tewari

**Affiliations:** 1 Diabetes and Endocrinology, Jai Clinic & Diabetes Care Center, Lucknow, IND; 2 Anatomy, Era's Lucknow Medical College and Hospital, Era University, Lucknow, IND; 3 Medicine, King George's Medical University, Lucknow, IND

**Keywords:** urinary-albumin-creatinine-ratio (uacr), body mass index (bmi), age, estimated glomerular filtration rate (egfr), chronic kidney disease (ckd), duration of diabetes, type 2 diabetes (t2d)

## Abstract

Introduction: Current evidence demonstrates that people with type 2 diabetes (T2D) are at a higher risk of developing chronic kidney disease (CKD) with greater morbidity and mortality. We, therefore, aimed to document and categorize demographic, anthropometric, and physiological risk factors of CKD in people with T2D in India. Additionally, we also attempted to evaluate the magnitude of each risk factor, namely age, duration of diabetes, HbA1c, and body mass index (BMI) in its etiology.

Methods: This observational, single-center, and cross-sectional study was performed at a diabetes care center in Lucknow, India. Out of a total of 504 eligible patients, we could get the required data from 435 patients. The following data were collected: demographic data, estimated glomerular filtration rate (eGFR), serum creatinine, urinary albumin creatinine ratio (UACR), and HbA1c levels. Appropriate statistical tests were applied.

Result: The 435 eligible people with diabetes had a mean age (SD) of 51 (±10.52) years; female 48.02%, duration of diabetes 7 (±5.4) years; HbA1c 8.6 (±2.3)% and eGFR values 80.2 (±26.6) mL/min/1.73m^2^ at the time of presentation. The eGFR values correlated negatively with age and duration of diabetes, and positively with increasing BMI. The Spearman correlation coefficient showed that clinical parameters such as age, duration of diabetes, and BMI have a weak, but statistically significant correlation with eGFR while eGFR did not correlate with HbA1c level in the study. Further, we did not find a correlation between eGFR and UACR.

Conclusion: In people with T2D, age and duration of diabetes are important risk factors for the development of CKD based on the eGFR. Hence, even in the absence of high UACR values, a low eGFR should prompt periodic monitoring to reduce the risk of progression of CKD, especially, in older people with long-standing T2D. Our study did not find HbA1c as a suitable tool to assess the CKD progression risk, but historical glycaemic control over longer periods revealed by sequential values of HbA1c over the duration of disease may correlate with the progression of CKD.

## Introduction

The twin epidemic of type 2 diabetes mellitus (T2D) and obesity is responsible for the rise in vascular complications [1]. Globally, its prevalence has increased from nearly 10% to more than 12% over two decades. One of the key complications of T2D is chronic kidney disease (CKD). In 2017, there were 697·5 million cases of CKD in the world, of which nearly 16% were in India [2]. Thus, CKD is one of the major causes of morbidity, affecting 20%-40% of people with diabetes [3]. The Third National Health and Nutrition Examination Survey (NHANES III) across 10 years, which determined cumulative mortality by diabetes and kidney disease status in over 15,000 participants, showed that those with kidney disease largely accounted for the increased mortality in people with T2D [4]. NHANES III showed, compared to people with diabetes without kidney disease, in people with both diabetes and kidney disease, the absolute risk difference of mortality was higher. Thus, the presence of kidney disease accounts for the increased mortality in people with T2D. In addition, the presence of CKD increases the risk of all-cause and cardiovascular mortality in people with diabetes [4]. There are several factors that can increase the risk for CKD namely age, gender, race/ethnicity, and family history along with hyperglycemia, hypertension, and cardiovascular disease. Elevated blood pressure, unhealthy dietary components, and consequent obesity are factors that aggravate the progression of CKD [1]. A meta-analysis determining associations of kidney disease parameters with mortality and end-stage renal disease (ESRD) in individuals with diabetes has shown that the addition of eGFR and urinary albumin-to-creatinine ratio (UACR) significantly improved the prediction of cardiovascular outcomes and mortality beyond conventional risk factors [5]. A new CKD classification, which includes both GFR and albuminuria stages, has been adopted to provide a more accurate assessment of renal and cardiovascular outcomes [6]. Low eGFR (GFR <60  mL/min/1.73 m^2^) or albuminuria, are the central features for the diagnosis of CKD as they are the best indicators of damage. Low eGFR may be present together with albuminuria in 12% of T2D subjects or alone in 11% as isolated low eGFR values [7]. In the current study, we aimed to document and categorize demographic, anthropometric, and physiological risk factors of CKD. Further, we also aimed to correlate each risk factor namely age, duration of diabetes, HbA1c, and BMI responsible for CKD in those diagnosed with T2D.

## Materials and methods

Study design

Data included all patients’ demographic records with respect to age, gender, BMI, Duration of Diabetes, HbA1c, eGFR level. This observational, cross-sectional study included patients with T2D people of either gender aged 18 years and above. In a previous study in India, the prevalence of CKD in patients with diabetes [8] was reported as 48.4%. Assuming a wastage factor of 25%-40% and a 5% absolute error and α error of 5%, the sample size of 504 was reached [9].

The study enrolled 504 patients with T2D during May 2020; we gathered: demographic data, serum creatinine (auto analyzer Erba Chem 5 Plus V2, TransAsia International), eGFR (CKD-EPI EQUATION), UACR (urilyser, Arkray Inc., Tokyo, Japan), and HbA1c levels (Affinion point of care device, Abbott Inc., Princeton, NJ, USA) in T2DM patients. Normoalbuminuria was defined as urinary albumin-to-creatinine ratio <30 mg/g in two or more urine samples. Microalbuminuria was defined as urinary albumin-to-creatinine ratio 30-299 mg/g in at least two urine samples. At follow-up, macroalbuminuria was defined by albumin-to-creatinine ratio >300 mg albumin per gram creatinine. Indicators of renal damage were an eGFR less than 60 mL/min/1.73m^2^ or albuminuria [10,11]. The study received clearance from the ethics committee of the institution (Registration no: ECR/241/Inst/UP/2019). We sought the written informed consent from all people enrolled in the study. People with a diagnosis of acute kidney injury, symptomatic urinary tract infection, history of hematuria, those who had undergone renal transplant or were on maintenance dialysis, or participants of any interventional study within the previous three months were excluded. Pregnant women were also excluded from the study.

Statistical analysis

Data were processed in an Excel sheet and analyzed using the SPSS software. Quantitative variables were presented as mean and standard deviation, if data were normally distributed, else as median and IQR. In order to determine the relationship between the eGFR and UACR with other variables and considering nonnormally distributed data, we used Spearman rank correlation or Spearman's Rho, which is a non-parametric test used to determine the strength of associations. The strength of a correlation was described as weak correlation (0.00 to 0.39), moderate correlation (0.40 to 0.69), and strong correlation (0.70 to 1.00).

Ethical statement

The study is being performed in accordance with ethical principles that are consistent with the declaration of Helsinki, international conference on harmonization of technical requirements for registration of pharmaceuticals for human use - good clinical practices and guidelines issued by Indian regulatory authorities for noninterventional studies.

## Results

We could get the required data from 435 people meeting the inclusion criteria from the Jai Clinic and Diabetes Care Centre, Lucknow. The age, gender, HbA1c, BMI, and duration of diabetes are as shown in Table 1.

**Table 1:** Demographic and laboratory characteristics of people with T2D T2D - type 2 diabetes

Parameter	N (%)
Age (years) (Mean±SD)	50.97±10.519
18-25	3 (0.6%)
26-35	41 (9.33%)
36-45	114 (26.19%)
46-55	140 (32.14%)
56-65	108 (24.6%)
66-75	26 (5.95%)
76-85	6 (1.19%)
Gender	
Male	227 (51.98%)
Female	209 (48.02%)
BMI (kg/m^2^) (Mean±SD)	25.718± 4.46
Underweight (<18.5)	16 (3.64%)
Normal (18.5 to <23)	178 (40.69%)
Overweight (23 and 24.9)	164 (37.65%)
Obese (≥ 25)	79 (18.02%)
Duration of diabetes years (Mean±SD)	7.2±5.36
<1 year	14 (3.01%)
1-5 years	193 (44.29%)
6-10 years	143 (32.67%)
11-15 years	52 (11.82%)
≥ 16 years	36 (8.22%)
HbA1c (Mean±SD)	8.549±2.27
eGFR (mL/min/1.73m^2^) (Mean±SD)	80.230±26.6230
Stages of chronic kidney disease	
Stage 1	156 (35.86%)
Stage 2	162 (37.22%)
Stage 3a	71 (16.17%)
Stage 3b	38 (8.72%)
Stage 4	4 (0.85%)
Stage 5	6 (1.28%)

**Figure 1:**
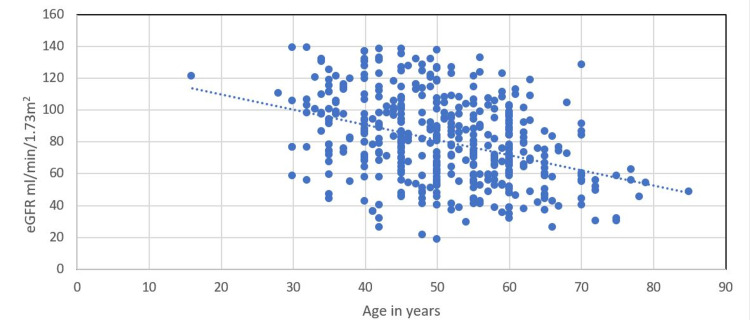
Relationship between eGFR (in mL/min/1.73m2) and age (in years) eGFR - estimated glomerular filtration rate

When the eGFR was plotted against age, the two were found to be inversely related (Figure 1).

**Figure 2:**
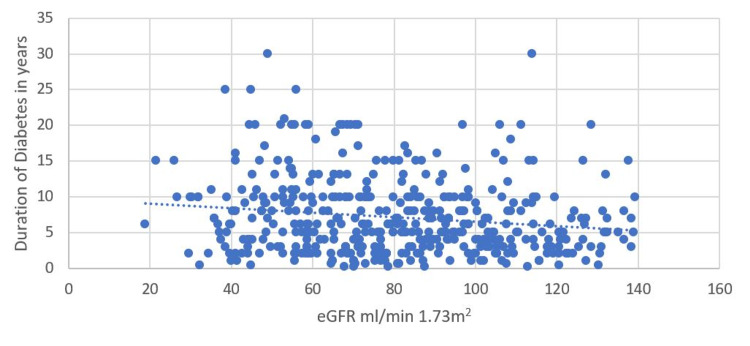
Relationship between eGFR and duration of diabetes (DoD) eGFR - estimated glomerular filtration rate

Similarly, the eGFR was found to be significantly decreasing with increasing duration of diabetes (p=0.003) (Figure 2).

**Figure 3:**
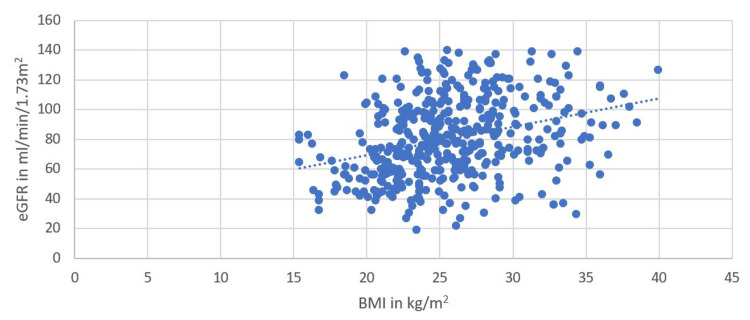
Relationship between eGFR (in mL/min/1.73m2) and BMI (in kg/m2) eGFR - estimated glomerular filtration rate, BMI - body mass index

On the contrary with increasing BMI, the eGFR was found to be increased (Figure 3).

Since we aimed to determine the relationship between the various variables and eGFR, we used Spearman rank correlation to determine the strength of their associations (Table 2) [12].

**Table 2:** Correlation analysis of eGFR values with the duration of diabetes (DOD), HbA1c, age, and body mass index (BMI) eGFR - estimated glomerular filtration rate

Correlation of eGFR	Correlation Coefficient	Sig. (2-tailed) P-value
Duration of diabetes	-0.144^**^	0.003
HbA1c	-0.057	0.239
Age	-0.365^**^	Less than 0.001
BMI	0.353^**^	Less than 0.001

Based on the data, though these are both statistically significant; age has a negative relationship, whereas BMI is positively correlated. The duration of diabetes is statistically significant and has a negative relationship with eGFR. Notably, the duration of diabetes of the people was between 1 and 5 years in 44.29% followed by 6-10 years in 32.67%. The stages of eGFR fairly matched with the Duration of diabetes, since the majority of people were in Stage 1 and Stage 2 of eGFR. While in nearly 20% the duration of diabetes was more than 10 years. Thus, the Spearman correlation coefficient shows that age, duration of diabetes, and BMI have a weak but statistically significant relationship with eGFR, while such an association with HbA1c was lacking in the current study.

While the majority of the people were normoalbuminuric (62.07%), almost half of people developed microalbuminuria (35.86%), but there were just 2.07% who developed macroalbuminuria (Table 3).

**Table 3:** Assessment of the presence of eGFR and albuminuria eGFR - estimated glomerular filtration rate

		Urinary albumin creatinine ratio				
		A1	A2	A3	Total	%
eGFR category	G1	139	18	1	158	36.32
	G2	86	81	1	168	38.62
	G3a	34	39	1	74	17.01
	G3b	11	17	3	31	7.13
	G4	0	1	3	4	0.92
	Total	270	156	9	435	
	%	62.07	35.86	2.07		

Several studies have shown that there is a significant degree of inconsistency between the development of albuminuria and renal impairment [13]. Therefore, albuminuria may not be consistent with renal impairment or decreased eGFR in the progression of CKD. Predictive factors for the onset of albuminuria and reduced eGFR suggest both common and discrete pathophysiological mechanisms in the development of these two indicators of CKD [13].

Among the 435 people who were included, the age in the majority of them fell between 36 and 65 years, and the gender distribution was approximately equally distributed. More than 55% of people in the study were overweight or obese. Most of them had a prior history of T2D for 1-5 years and a quantifiable 32.6% were within the range of 6-10 years.

If we compare the proportion of people according to the BMI and duration of diabetes groups, 78.34% were normal or overweight diabetics and the duration of diabetes was between 1 and 10 years in 76.96% of people. These data correspond to nearly 72.34% of people in normal or mildly reduced eGFR.

Using Spearman’s rho analysis, we also found that the results of our study underscore a quantifiable association of renal function cumulatively with “age” and “duration of diabetes.” Among the parameters we evaluated, age, and duration of diabetes were negatively associated and BMI positively associated with declining eGFR.

## Discussion

Largely, the prevalence of CKD increases with age in men and women subjects. Age-related renal abnormalities predispose older people to an increased risk of CKD. As the aging population is growing, the prevalence of older people with CKD is expected to rise [14]. A recent study in the Chinese urban population found that the prevalence of reduced eGFR was much higher in women over 40 years than in their male equals, and the overall prevalence was higher with older age in both gender [12]. The current study corroborates these findings, which found a decline in eGFR with increasing age. Further, all the diabetic people aged more than 66 years were found to be in eGFR stage 3a (<60 mL/min/1.73m^2^).

A plethora of evidence exists demonstrating obesity as a risk factor for reduced eGFR or CKD. The Framingham study cohort showed that BMI could predict reduced kidney function regardless of the presence of comorbidities like diabetes or hypertension [15]. Nevertheless, our results are not aligned with the Framingham cohort study. But BMI as a predictor of kidney disease may not a robust parameter because it cannot differentiate between the body muscle and the body fat [16]. Studies have also found that high BMI is associated with greater survival in people on maintenance hemodialysis, which suggests that it is the gross muscle mass and not total body weight that provides protection [17]. Since BMI does not account for the difference between these, it might not be used as a dependable indicator of reduced eGFR or decline in renal function, particularly for T2D people [16].

The results of observational studies and a large cohort of 27,029 people with T2D followed-up at over 200 centers showed that elderly people and those with longer duration of diabetes have a 49% risk of developing eGFR <60  mL/min/1.73 m^2^ [6,18]. Another cross-sectional observational study showed that people who developed CKD had longer diabetes history (mean duration of 13 versus 9 years) [19]; the current study aligns with these findings. Previous studies have observed that Stage I, GFR is either normal or increased and remains so for ~5 years from the onset of diabetes; Stage II, begins approximately two years later and most people remain in this stage for life [20]. These data support the findings of the current study because we observed a similar trend. Stage 3, which indicates the first clinically identifiable sign of glomerular damage, generally occurs 5 to 10 years after the onset of the disease and in our study, nearly 16% of people were in Stage 3 proportionate to those in the duration of diabetes of more than 10 years [19]. Those in 16 years and above (~8%) were more likely to be in Stage 3b and above and the proportion (9.85%) corresponds to those in Stage 3b up to Stage 5.

Further, the HbA1c negatively correlated with eGFR but it was not statistically significant, since it is an indicator of short-term blood glucose control. A recent study showed that in a referred population of established CKD, higher HbA1c was not associated with a higher risk of ESRD or death rather intensive blood glucose control was probably owing to the impaired clearance of drug used, and impaired counter-regulatory response, and an increased burden of co-morbidity [21].

Limitations

This study has experienced certain limitations since it was a single-center study not typical of the general population, leading to difficulty to take a broad view in patients with T2D mellitus. A larger study would be required to provide more confirmatory findings. Clinical HPLCs are considered the current gold standard for HbA1c testing due to their high reproducibility and specificity but we could not measure HbA1c due to the highly technical maintenance factors required for measurement. This study could not collect rele­vant information on the waist-to-hip ratio, which would have been a more robust indicator for fat and muscle distribution in patients with CKD rather than BMI. Further, since this is a cross-sectional study, the results should be in­terpreted with caution due to the typical causality issue. However, it is noteworthy that this study has captured data from a fairly large number of patients to obtain clinically relevant results.

## Conclusions

The present study provides consistent and recent epidemiological data showing an inverse association of eGFR with age and duration of diabetes, while it correlated with BMI among people with T2D. This study highlights that age and duration of diabetes are key parameters associated with declining renal functions in people with T2D in India. However, the absence of urinary albumin should not be considered an indicator of normal renal function, since the pathophysiology of both eGFR and albuminuria involve discreet mechanisms too. Hence, even in the absence of higher UACR, a low eGFR should prompt periodic monitoring to reduce the risk of progression to higher stages of CKD especially in older people and in those with long-standing. The study was conducted at an urban center, with people receiving optimum diabetes care. It may not be a true representation of the general population. Epidemiological studies in population settings in terms of prospective cohort studies may further strengthen the association.
